# Population Genetic Structure and Selection Signature Analysis of Beijing Black Pig

**DOI:** 10.3389/fgene.2022.860669

**Published:** 2022-03-24

**Authors:** Wenjing Yang, Zhen Liu, Qiqi Zhao, Heng Du, Jian Yu, Hongwei Wang, Xiance Liu, Hai Liu, Xitao Jing, Hongping Yang, Guohua Shi, Lei Zhou, Jianfeng Liu

**Affiliations:** ^1^ College of Animal Science and Technology, China Agricultural University, Beijing, China; ^2^ Beijing Heiliu Stockbreeding Technology Co.,Ltd, Beijing, China

**Keywords:** whole-genome sequencing, beijing black pig, genetic diversity, selection regions, candidate genes

## Abstract

Beijing Black pig is an excellent cultivated black pig breed in China, with desirable body shape, tender meat quality and robust disease resistance. To explore the level of admixture and selection signatures of Beijing Black pigs, a total number of 90 individuals covering nine pig breeds were used in our study, including Beijing Black pig, Large White, Landrace, Duroc, Lantang pig, Luchuan pig, Mashen pig, Huainan pig and Min pig. These animals were resequenced with 18.19 folds mapped read depth on average. Generally, we found that Beijing Black pig was genetically closer to commercial pig breeds by population genetic structure and genetic diversity analysis, and was also affected by Chinese domestic breeds Huainan pig and Min pig. These results are consistent with the cross-breeding history of Beijing Black pig. Selection signal detections were performed on three pig breeds, Beijing Black pig, Duroc and Large White, using three complementary methods (F_ST_, *θπ*, and XP-EHH). In total, 1,167 significant selected regions and 392 candidate genes were identified. Functional annotations were enriched to pathways related to immune processes and meat and lipid metabolism. Finally, potential candidate genes, influencing meat quality (*GPHA2, EHD1*, *HNF1A*, *C12orf43*, *GLTP*, *TRPV4*, *MVK*, and *MMAB*), reproduction (*PPP2R5B* and *MAP9*), and disease resistance (*OASL*, *ANKRD13A*, and *GIT2*), were further detected by gene annotation analysis. Our results advanced the understanding of the genetic mechanism behind artificial selection of Beijing Black pigs, and provided theoretical basis for the subsequent breeding and genetic research of this breed.

## Introduction

From early domestication to modern breeding practices, artificial selection for agricultural economic traits has shaped the genomes of domestic pigs and led to many breeds and populations worldwide (Gouveia et al., 2014). Under positive selection pressure, the frequencies of favorable alleles would increase, and it would show an unusual long-range linkage disequilibrium (LD) with a high population frequency (Smith and Haigh, 1974). Signatures of selection in the genome have been used frequently to understand the relationships between genotype and phenotype in pigs. For instance, strong selection signatures were found at three loci which were related to morphological changes in the domestic pigs using whole-genome resequencing (Rubin et al., 2012); evidence of artificial selection of lean muscle mass, fertility and immunization traits were revealed in Duroc pigs (Ma et al., 2018).

Beijing Black pig (BJB), which is a typical composite black pig breed in China, is best known for its perfect combination of the characters of Chinese native pig breeds (i.e., superior meat quality, strong resistance to disease and desirable reproduction performance) and commercial pig breeds (i.e., fast growth rate, high lean meat rate and feed conversion efficiency). For example, this breed is renowned for its meat quality and high intramuscular fat content in pork with an average value of ∼3.11% as compared to less than 2% of commercial pig breeds (Zhang et al., 2018).

Beijing Black pigs have a wide range of breed compositions. According to the breeding history of BJB, it was cross-breeded from multiple Chinese native pigs and foreign commercial pig breeds, including Chinese Northern pig breeds, Chinese Southern pig breeds, Huanghuaihai pigs, Large White, and Berkshine, etc. After many years of systematic genetic improvement programs, BJB was certificated as a new pig breed by the Ministry of Agriculture of China in 1982. In recent years, consumers are increasing focus on health and food quality, and prefer pork with higher quality and better flavors. Beijing Black pig has undoubtedly contributed to offering high-quality pork in the present-day market. However, to date, only a few studies have focused on BJB. A genome-wide association study of vertebral and teat number was performed on 891 BJB by Illumina Porcine 50 K BeadChip, and several quantitative trait loci (QTL) were identified (Niu et al., 2021). Candidate genes for skeletal muscle growth and meat quality of Beijing Black pigs were found via the RNA-seq method (Hou et al., 2021). However, the genetic basis of the characteristics of BJB, particularly at the genomic level, remains largely unknown. Specifically speaking, it is still unknown to what extent the breed contribution of Beijing Black pigs is influenced by commercial breeds and Chinese local breeds. Also, it is essential to detect selection signatures and genes related to the biological processes for the economic important traits of Beijing Black pigs, which have undergone decades of intense artificial selection.

In this study, we used whole-genome resequencing data of BJB, together with eight additional pig breeds, representing potential breed origin of BJB. These eight pig breeds including Huainan pig (HN) and Mashen (MS) pig (from Huanghuaihai district); Lantang pig (LT) and Luchuan pig (LC) (from southern China); Min pig (MIN) (from northeast China); Duroc (DU), Landrace (LD), and Large White (LW) (representing three major commercial pig breeds). We conducted a comprehensive analysis of phylogenetic relationships and genetic diversity among these pig breeds, then three selection signature detection methods were performed to identify genomic regions under selection and potential candidate genes in BJB. Our findings enable to better understand the Beijing Black pig’s genome characteristics, and provide novel insights for developing breeding strategies and germplasm conservation in the near future.

## Materials and Methods

### Sample Collection and Sequencing

A total number of 59 samples, including 22 Beijing Black pigs, 22 Duroc, five Lantang pigs, five Mashen pigs, and five Huainan pigs, were sequenced in this study (Supplementary Table S1). Genomic DNA was extracted from pig ear tissue using the Qiagen DNeasy Tissue Kit (Qiagen, Germany). Then, DNA integrity and purity were verified using agarose gel electrophoresis and A260/280 ratio. All samples were constructed from Illumina DNA library (paired end, 2 × 150 bp) and sequenced using Illumina HiSeq 2000 sequencing system. In addition, genomic data of 5 Min pigs, five Luchuan pigs, five Landrace, and 16 Large White pigs were downloaded from public domain (Supplementary Table S1). In total, whole genome sequencing data of 90 pig samples of nine breeds were analyzed in this study. The raw resequencing reads were filtered using fastp v0.20.1 (Chen et al., 2018), by removing reads containing adapters, low-quality reads with >30% base (quality value ≤ 20, or N bases) and low-quality 3’ end reads with base quality scores of ≤20.

### Variant Calling and Annotation

Clean sequencing reads were subsequently mapped to the pig reference genome Sus scrofa 11.1 using Burrows-Wheeler Aligner (BWA) v0.7.17 (Li and Durbin, 2009) with default settings. Genome Analysis Toolkit (GATK) v3.8 (Mckenna et al., 2010) was used for SNP calling. HaplotypeCaller and GenotypeGVCFs modules in GATK were jointly used to call variation. Intermediate genomic (gVCF) files were generated using the “-ERC GVCF” mode in HaplotypeCaller. Then, joint genotypes were determined using GenotypeGVCFs. High-quality SNPs, with Quality score>30, MQ RMS mapping quality >20, DP > 5, coverage >30% and minor allele frequency (MAF) > 0.01, were kept with vcftools v0.1.16 for the following analysis (Danecek et al., 2011). Then, the dbSNP database (Sherry et al., 2001) was used to identify the novel genetic variations. Finally, ANNOVAR v2019Oct24 was used to conduct gene-based or region-based annotation processing for the filtered variants (Wang et al., 2010), and the corresponding gene annotation file was downloaded from the Ensembl database (https://asia.ensembl.org/index.html).

### Population Structure Analysis

A common subset of 22,112,606 SNPs resulted from the above procedures was used to infer the genetic structure of these nine pig breeds. Genetic distances between individuals and breeds were calculated using an identity-by-state (IBS) similarity matrix via Plink v1.9 software (Purcell et al., 2007). Neighbor-joining (NJ) phylogenetic tree was built based on IBS distance matrix using the PHYLIP v3.698 (Felsenstein, 1993). After that, the NJ tree was visualized via Figtree v1.4.3 (http://tree.bio.ed.ac.uk/software/figtree/). Principal component analysis (PCA) was performed by GCTA v1.91.7 (Yang et al., 2011), in which the genetics matrix was firstly generated using “—make-grm” option, and then the first three principal components were calculated with “—pca3” option. Finally, the PCA plot was plotted using the R language. Moreover, the population ancestry of these breeds was inferred by ADMIXTURE v1.3.0 (Alexander et al., 2009). The optimum number of ancestral clusters K was estimated with a five-fold cross validation procedure. The ancestral clusters number K were tested from 2 to 9, using the plots of ancestry compositions of the tested breeds by a R package “Pophelper” (Francis, 2017).

### Analysis of Genome Diversity and Inbreeding

The F-statistic (F_ST_) is a population differentiation index based on genetic polymorphism data (Holsinger and Weir, 2009). Vcftools v0.1.16 was implemented to evaluate F_ST_, and an F_ST_ matrix of size 9 × 9 were further obtained, which was graphically represented by heatmap *via* corrplot R package (Wei et al., 2017). Running of homozygosity (ROH) fragments of each individual were determined using Plink v1.9. Then, the average number of ROH fragments per breed was classified into seven categories: 100–200 kb, 200–300 kb, 300–400 kb, 400–500 kb, 500–600 kb, 600–700 kb, and >700 Kb. ROH-based inbreeding coefficient (F_ROH_) was measured by the ratio of the total length of ROH to the length of autosomes (2.27 Gb in this study) (Mcquillan et al., 2008).

### Selection Signature Detection of the Beijing Black Pig

Three methods for genomic selection signature detection, including population differentiation coefficient (F_ST_), polymorphism levels statistic (*θπ*) and cross-population extended haplotype homozygosity (XP-EHH), were performed to detect the genomic regions under selection in Beijing Black pigs by comparing with Duroc and Large White. The F_ST_ and *θπ* (DU or LW/BJB) between the Beijing Black pigs and the other two breeds were calculated with vcftools v0.1.16 using 100 kb windows with 10 kb steps among genomes. Then, *θπ* (DU or LW/BJB) was log2-transformed. The XP-EHH statistic was designed to detect ongoing or nearly fixed selection signatures by comparing haplotypes from two populations (Sabeti et al., 2007). XP-EHH values were estimated using Selscan v1.3.0 (Szpiech and Hernandez, 2014). Then, the XP-EHH value of each SNP was normalized. Extremely high values in the 5% right-tail of each method were empirically selected as potential candidate regions under positive selection.

We also estimated allele frequencies of single-nucleotide variants (SNV) with a genome scan for each pig population, and measured the absolute allele frequency difference (ΔAF) for comparing different breeds. The ΔAF per SNV between the Beijing Black population and the other two populations was calculated using the formula: ΔAF = abs (AltAF_BJB_-mean (AltAF_Du_ + AltAF_LW_)) (Zhao et al., 2018).

### Genome Annotation and Enrichment Analysis

Genes in these selection regions were identified through the ensembl database Sus scrofa 11.1 assembly.

(http://ftp.ensembl.org/pub/current_gtf/sus_scrofa/Sus_scrofa.Sscrofa11.1.105. gtf.gz). To further explore the potential biological significance of genes within these candidate regions, Gene Ontology (GO) terms and Kyoto Encyclopedia of Genes and Genomes (KEGG) pathway enrichment analyses were carried using KOBAS (Xie et al., 2011). Finally, published pig QTLs were downloaded from pig QTLdb database (Hu et al., 2022) to identify the QTL overlap with the candidate regions in our study.

## Results

### Genomic Variant Identification in Beijing Black Pig

Whole-genome resequencing of 59 individuals from five pig breeds (BJB, DU, HN, LT, and MS) generated a total size of 2,196 Gb raw paired-end reads. To fully explore the breed origin of BJB and accurately detect genomic footprints left by selection, whole genome resequencing data of another 31 samples of LD, MIN, LU and LW pigs (Supplementary Table S1) were downloaded from the public available database. After quality control, genomes of the above 90 pigs were aligned against the sus scrofa 11.1 reference genome using the BWA v0.7.17, resulting an average depth of 18.19 folds (Supplementary Table S1).

**FIGURE 1:**
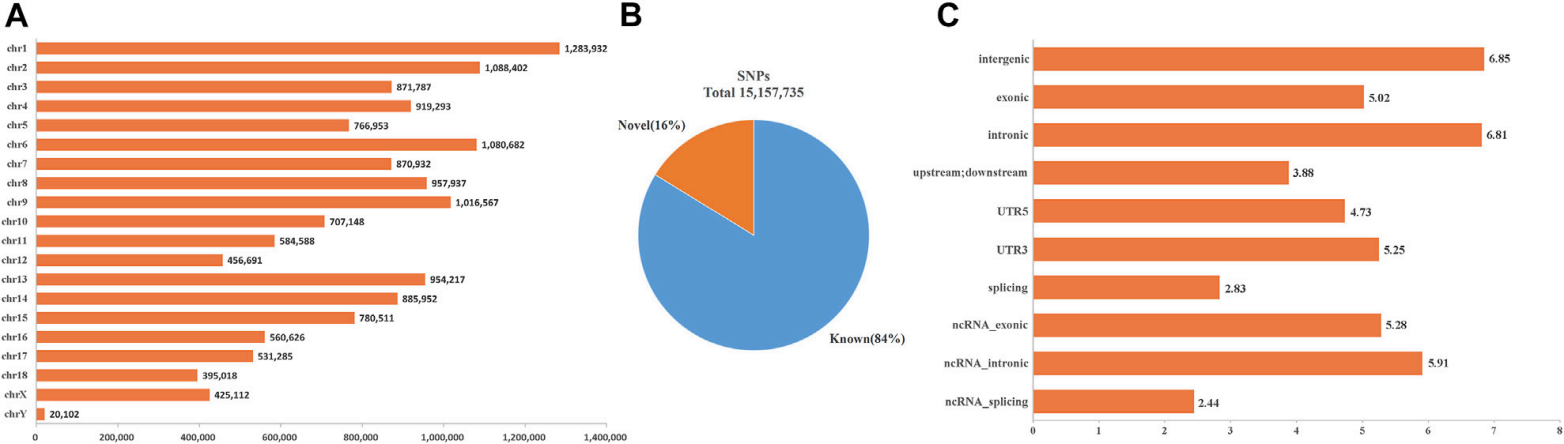
SNV characteristics of Beijing Black population. **(A)** Genome-wide distribution of detected SNVs on 19 chromosomes for the Beijing Black pigs. *X*-axis represents the number of SNVs. *Y*-axis represents 18 autosomes and X chromosome. **(B)** The percentage of Beijing Black SNVs within the dbSNP database. **(C)** Genome-wide annotation of Beijing Black genetic variations. *X*-axis represents the number of genetic variations (log10) within various functional regions. *Y*-axis represents various functional regions.

After variants calling and subsequent stringent quality control, a total number of 15,157,735 SNVs were identified in the BJB population with high quality (Figure 1A), of which 2,458,021 SNVs (16%) were considered as novel based on their absence in the pig dbSNP database (Figure 1B). Then, all detected SNVs in BJB were annotated using the gene annotation file downloaded from the Ensembl database. As it was expected, the largest number of SNPs was found in intergenic regions (46.42%) and introns (43.07%). Only 0.70% of them were located in exonic regions, including 61,674 synonymous and 42,859 non-synonymous mutations (Figure 1C, Supplementary Table S2). These potential functional SNPs provide valuable genetic resources for exploring the genetic structure and selective characteristics of BJB.

### Phylogenetic Relationships and Population Structure Analysis

**FIGURE 2:**
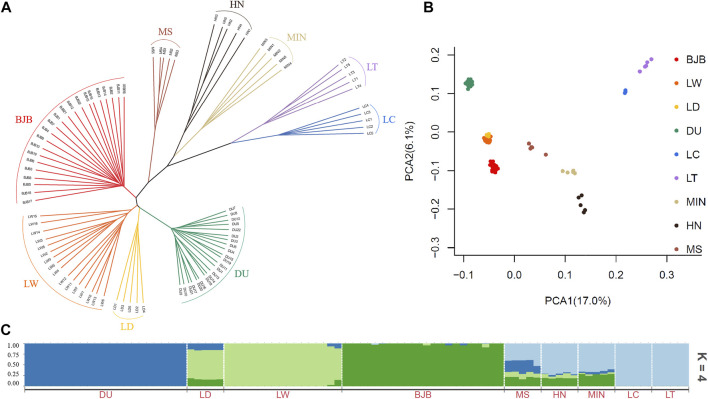
Phylogenetic relationship and population structure of Beijing Black and other eight breeds tested in this study. BJB, Beijing Black pig; HN, Huainan pig; MS, Mashen pig; LT, Lantang pig; LC, Luchuan pig; MIN, Min pig; DU, Duroc; LD, Landrace; LW, Large White. **(A)** Neighbor-joining phylogenetic tree constructed from SNV data among nine populations. **(B)** Principle component analysis for the first two PCs of 90 pigs. **(C)** ADMIXTURE analysis with four presumed ancestral groups (K = 4).

To assess the phylogenetic relationship among the nine pig breeds, an identical-by-state (IBS)-derived NJ tree were conducted for all tested individuals (Figure 2A). The tree showed that individuals of the same breed generally were clustered together, which signified that they possessed unique breed identities. The NJ tree revealed a clear divergence between modern commercial breeds and Chinese indigenous breeds, as the three modern commercial breeds (DU, LD, LW) formed a separate cluster while all the Chinese local breeds defined a large new clade. Interestingly, we noted that BJB were located at intermediate positions between these two major clades, which was consistent with its breeding history.

The result of PCA analysis was consistent with the above NJ clustering pattern (Figure 2B). The first two PCs explained 17.0% and 6.1% of the total variation, respectively. It was obvious that BJB had closest genetic relationships to the commercial breeds including DU, LD and LW pigs, followed by Chinese indigenous pigs MS, MIN and HN, whereas LT and LC pigs were least related to BJB pigs. This may indicate that in the breeding history of BJB, more Western breeds such as Large White pigs were more heavily used in the crossbreeding of BJB than the Chinese indigenous pigs.

To investigate admixture levels among all tested breeds, we performed the ADMIXTURE analysis assuming ancestral number K from 2 to 9 (Figure 2C, Supplementary Figure S1). According to our results, K = 4 represented the optimal number of assumed ancestors by cross-validation error test (Supplementary Figure S1). In this scenario, Beijing Black pigs and Western commercial breeds were differentiated, and a certain proportion of Western ancestries and Chinese native pig breeds were still evidenced in BJB. BJB has formed a unique genetic structure after multiple generations of breeding, which indicates that it can be used as an independent genetic resource.

### Genetic Diversity of Beijing Black Pig

**FIGURE 3:**
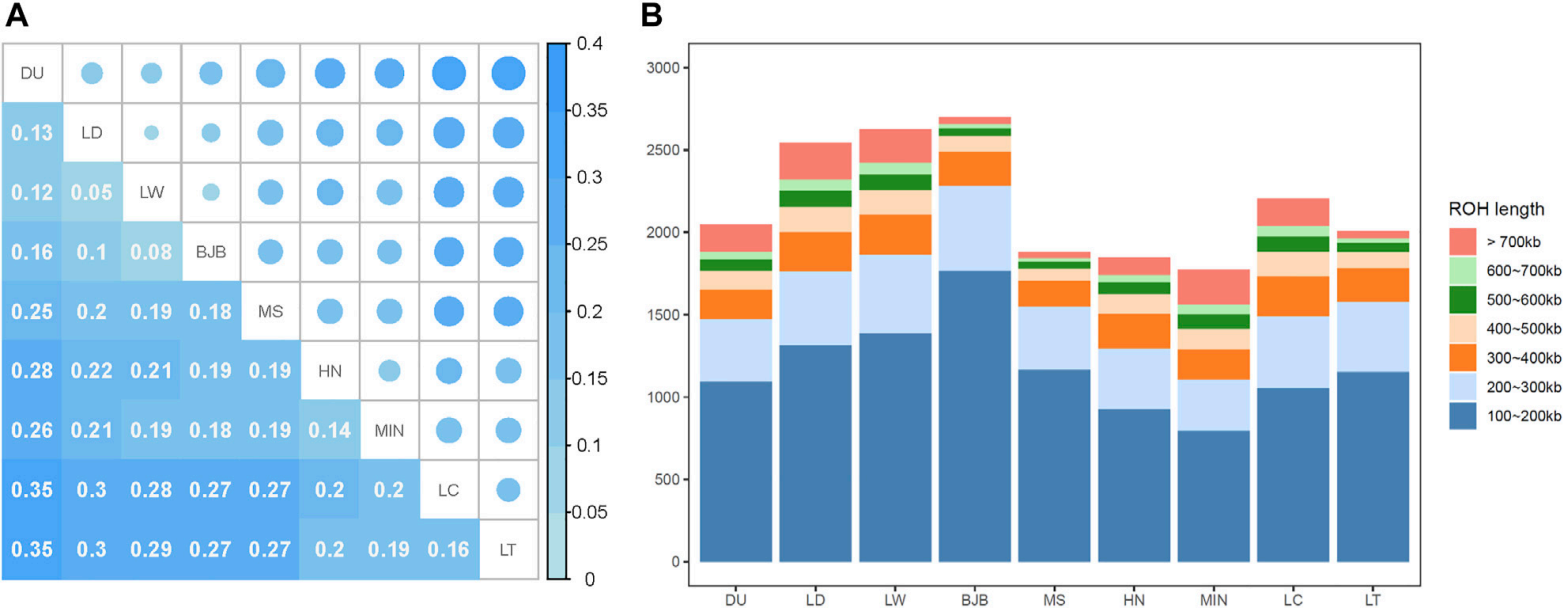
Genetic diversity of Beijing Black pigs. **(A)** Heatmap of F_ST_ distance between breeds. **(B)** The average number of ROH fragments of each breed. The number of ROH was classified into seven categories.

The genetic diversity between pairs of pig populations was investigated with the F_ST_ index (Figure 3A). The genetic differentiation between BJB and other lean commercial pig breeds (ranged from 0.08 to 0.16) were less than that between BJB and Chinese local pig breeds (ranged from 0.18 to 0.27), and the genetic differentiation between BJB and Large White was the lowest (0.08).

We calculated the inbreeding coefficients F_ROH_ based on ROH. In general, F_ROH_ of commercial pig breeds (ranging from 0.2696 to 0.3464) were higher than that of Chinese native pig breeds (ranging from 0.1825 to 0.2974). F_ROH_ of BJB (0.1906) was the fourth lowest among these nine breeds. The results showed that the existing breeding program could effectively avoid inbreeding of Beijing Black pigs to a certain extent. ROH fragments were then subdivided into seven categories (Figure 3B). We found that Beijing Black pigs had the highest number of ROHs, and ROHs of 100-200 Kb accounted for more than 60% of total ROHs in BJB. This may suggest that inbreeding events did not occur in recent generations in BJB or there may be a high proportion of inbreeding events in the first few generations of BJB.

### Selection Signature Detection of the Beijing Black Pig Population

**FIGURE 4:**
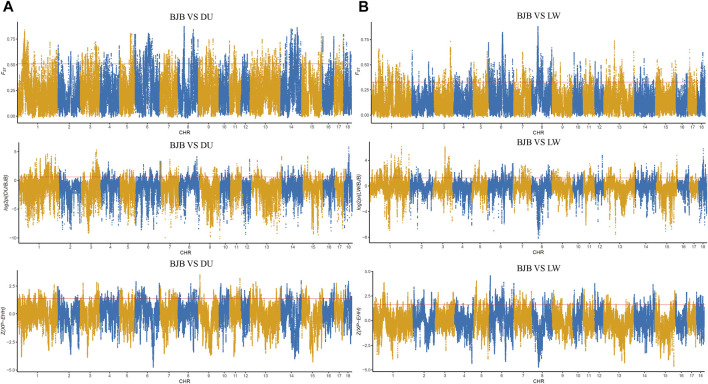
Genome-wide distribution of selection signatures detected by F_ST_, *θπ*, and XP-EHH on 18 chromosomes from top to bottom. *X*-axis represents 18 autosomes, and *Y*-axis represents statistic values of each method. The *θπ* values are log2 normalized, and the XP-EHH values are standardized. Red line displays the threshold level of 5%. **(A)** Global distribution of statistic values of three methods between Beijing Black pigs and Duroc. **(B)** Global distribution of statistic values of three methods between Beijing Black pigs and Large White.

**FIGURE 5:**
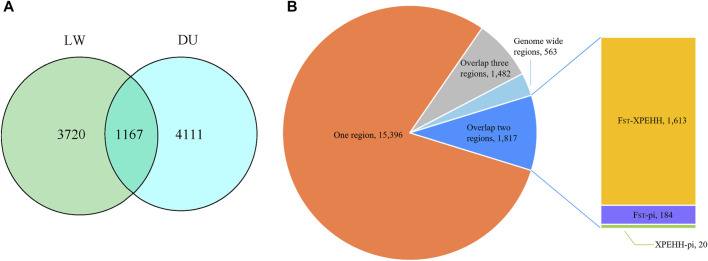
**(A)** Venn diagram shows the overlap in the number of candidate regions detected by different populations. The left circle represents the numbers of candidate regions detected by more than two methods between Beijing Black pig and Large White. The right circle represents the numbers of candidate regions detected by more than two methods between Beijing Black pig and Duroc. **(B)** The number of SNPs with high ΔAF in each region between Beijing Black pig and other two breeds.

In order to explore the genomic evidence related to breed features of Beijing Black pigs, we further compared the genomic signatures of BJB with two typical commercial pig breeds at population level (i.e., 22 Duroc and 16 Large White pigs). Three complementary methods (i.e., genetic differentiation (F_ST_); polymorphism level (*θπ*); cross-population extended haplotype homozygosity (XP-EHH)) were used to investigate genome-wide selection signals. In order to reduce false positive candidate regions, regions meeting the top 5% threshold in at least two methods were selected as selected regions. The genome distributions of candidate regions detected by different methods of BJB to Duroc and Large White pigs were shown in Figure 4. There were 5,278 selected regions (threshold, 5%; F_ST_, 0.508214; θπ ratio, 0.599414; XPEHH: 1.384220, Supplementary Table S4–6, Supplementary Figure S2) between BJB and Duroc population, whereas 4,887 selected regions (threshold, 5%; F_ST_, 0.328,283; *θπ* ratio, 1.234,365; XPEHH: 1.610,046, Supplementary Table S7–9, Supplementary Figure S2) between BJB and Large White. Meanwhile, there were 1,167 candidate regions overlapped between the above two groups of selected regions. (Figure 5A, Supplementary Table S10).

Since highly differentiated SNVs across populations are more likely to occur in the vicinity of the selected regions (Carneiro et al., 2014), we further compared the alternate allele frequency of all identified SNVs in BJB with those in the other two populations. Then, the absolute allele frequency difference ΔAF = abs (AltAF_BJB_-mean (AltAF_DU_ + AltAF_LW_)) were calculated to assess the potential selective sweeps of BJB. Significant enrichment of high-ΔAF SNVs (>0.8) within the identified sweep regions were observed, particularly the overlapping regions identified using both F_ST_ and XPEHH methods (Figure 5B). This reflected the fact that the highly differentiated population SNVs were actually associated with artificial and natural selection.

### Gene Annotation and Functional Analysis

We annotated 392 genes in the 1,167 candidate regions (Supplementary Table S11). Considering the deficiency of functional annotation of pig genome, candidate genes were transformed into human homologous genes using the Ensembl database. The gene annotation and pathways analysis showed that, genes were found to be significantly (FDR<0.05) enriched in 110 GO terms and 15 KEGG pathways (Supplementary Table S12). Some important pathways were found in the GO analysis, including lipid binding, glycerol metabolic process, adaptive thermogenesis, etc. In the KEGG analysis, most of significant pathways were related to disease (9 out of 15) and metabolic (2 out of 15), including papillomavirus infection, PI3K-Akt signaling pathway, and fat digestion and absorption, etc.

To identify publicly reported QTLs overlapped with these candidate regions, a total number of 34,342 QTLs of 708 different traits were downloaded from the Pig QTLdb database (Release 46, 27 Dec 2021). In total, there were 1,960 porcine QTLs (Supplementary Table S13) which were identified to be located within or overlapping with these 1,167 candidate regions. Notably, 1,287 (65.7%) QTLs were associated with meat and carcass traits, suggesting selection for meat traits during the breeding of BJB.

To further narrow down the candidate genes, we selected the 43 candidate regions that were detected in all three methods and overlapped in BJB compared with both Large White and Duroc (Supplementary Table S14). Twenty-five functionally important genes relevant to the excellent phenotype of BJB were identified, such as meat quality (*GPHA2, EHD1*, *HNF1A*, *C12orf43*, *GLTP*, *TRPV4*, *MVK*, and *MMAB*), reproduction (*PPP2R5B*), and disease resistance (*OASL*, *ANKRD13A*, *GIT2*). These genes were located on chromosomes 2, 6, and 14 respectively (Supplementary Table S15).

Through comparison of gene frequencies between BJB and the other two representative breeds, a total of 22 candidate BJB-specific SNVs were identified in exonic regions with the criteria of AF_BJB_ > 95% and AF_non-BJB_ < 5%, including seven nonsynonymous SNVs found in three genes (*ENSSSCG00000008892*, *ENSSSCG00000036417*, and *MAP9*, Supplementary Table S16). The three genes are located in the 42–43 Mb region of chromosome eight and overlapped with the F_ST_ candidate region of BJB compared to Large White and Duroc pigs. Therefore, it is speculated that this region of BJB is a selection region compared with the other two breeds.

## Discussion

In this study, the whole genome resequencing of 22 Beijing Black pigs, 25 Chinese local pigs and 43 commercial pigs representing nine breeds were performed. Phylogenetic analysis, principal component analysis and population structure analysis showed that resequencing data could effectively distinguish BJB from commercial pig breeds and Chinese local pig breeds. Long-term and intensive artificial selection resulted in great differences in genome and breed specificity of Beijing Black pigs. Population genetic differentiation (F_ST_) of Beijing Black pigs and other populations ranged from 0.10 to 0.27, which showed that Beijing Black pigs were more genetically similar to the commercial pig breeds than Chinese local pigs. However, Beijing Black pig still retains a small amount of genetic components of Huainan pig and Min pig (Supplementary Figure S1), which is consistent with the breeding history of Beijing Black pig.

Studies have demonstrated that inbreeding coefficient estimated by F_ROH_ is more accurate than that estimated by pedigree (Purfield et al., 2012), so we calculated inbreeding coefficient F_ROH_ in different pig populations. The results showed that the inbreeding coefficient of different pig populations ranged from 0.1825 to 0.3464, among which the inbreeding coefficient of BJB was 0.1906, which was relatively low among all tested populations. This indicates that although the core population size of BJB is small, the existing breeding programs effectively avoid inbreeding to a certain extent. The length of the homozygous fragment depends on the generational distance between the two individuals to a common ancestor. The shorter the homozygous fragment is, the farther the common ancestor is (Purfield et al., 2012). We found that the length of ROH fragments of Beijing Black pigs mainly concentrated in 100 KB ∼ 200 KB. It is speculated that there was a high proportion of inbreeding behavior in the early generations of breed formation (Ceballos et al., 2018).

Beijing Black pigs have characteristics of excellent meat quality, early puberty, and great disease resistance due to the intensive artificial selection for many years. Therefore, there must be selection signatures on the Beijing Black genome. Windows with simultaneously high F_ST_ values, significantly high θπ ratios and high XPEHH values (5% right tail) were selected among populations. In total, there were 1,167 selected regions detected by the across-breeds comparisons, and 382 candidate genes were further identified within these regions. Functional enrichment analyses revealed that these selected genes may play an important role in meat quality, reproduction, and immune process.

We detected a list of genes putatively under selection that are functionally related to BJB breed features, such as *GPHA2*, *EHD1*, *HNF1A*, *MVK*, and *MMAB* for meat quality. *GPHA2* is a cystine forming polypeptide and a subunit of the dimer glycoprotein hormone family. The region near *GPHA2* was significantly associated with the meat tenderness in the genome-wide association analysis of Australian beef cattle (Bolormaa et al., 2011). *EHD1* regulates the recycling of various receptors from the endocytic recycling compartment to the plasma membrane (Kieken et al., 2007). GWAS in pigs found *EHD1* was significantly associated meat to fat ratio (MFR) trait (Falker-Gieske et al., 2019), and *EHD1* knockout mice demonstrated that *EHD1* regulates cholesterol homeostasis and lipid droplet storage (Naslavsky et al., 2007). *HNF1A* encodes the protein which is a transcription factor required to express several liver-specific genes and plays an important role in glucose activation, insulin secretion regulation, and lipid metabolism (Pearson et al., 2007). Studies have found that polymorphism of *HNF1A* is significantly correlated with psoas muscle area, backfat thickness, fat content and muscle glycogen metabolism in Yorkshire and Berkshire pigs (Murphy et al., 2007; Fan et al., 2010). *HNF1A* is linked to several Quantitative Trait Loci (QTL) regions related to meat quality on chromosome 14, and is considered as an important candidate gene for meat quality traits (Uemoto et al., 2012; Kayan et al., 2013). Besides, there is a non-synonymous mutation in the *HNF1A* (Supplementary Table S17). *MVK* gene regulates cholesterol biosynthesis and terpenoid skeleton biosynthesis through SREBP. It was found that the expression of this gene is related to backfat thickness in pig in previous analyses (Kumar et al., 2019). Interestingly, five non-synonymous mutations were found within the *MVK* (Supplementary Table S17). *MVK* and adjacent *MMAB* gene is enriched in the same metabolic pathway. A study in mice reported that *MMAB* and *MVK* share a conserved promoter region and are both affected by sterol regulatory factor binding protein 2, indicating the two genes may share the same function.

Several candidate genes relating to reproduction were also detected, including *PPP2R5B* and *MAP9*. *PPP2R5B*, a regulatory subunit of PP2A is responsible for the dephosphorylation and inactivation of Akt protein (Beg et al., 2016). Studies have shown that *PPP2R5B* played a significant role in maintaining the fertility of boars at high temperatures (Hu et al., 2019), and this gene was expressed stably throughout lactation in sows (Tramontana et al., 2008). *MAP9* involved in mitotic spindle formation (Saffin et al., 2005). *MAP9* is a key factor in the early stage of development in zebra fish studies. Knockdown or overexpression of *MAP9* gene in zebra fish would lead to defects in early embryo development (Venoux et al., 2008).

The function of *ANKRD13A* was speculated to be immune-related. It regulated the K63 ubiquitination form of epidermal growth factor receptor (Tanno et al., 2012) and the endocytosis of B cell antigen receptor by interacting with endocytosis in humans (Satpathy et al., 2015). *OASL*, known inducers of antiviral activity, was found up-regulated of pigs infected with Classical swine fever virus (CSFV). Functional annotations showed that two non-synonymous mutations occurred within *OASL* (Supplementary Table S17). Therefore, our data might provide insight into the role of candidate genes in the immunity of BJB.

Overall, we comprehensively evaluated the genetic relationship and genetic diversity of Beijing Black pigs with commercial pig breeds and Chinese local pigs, providing new insights into the historical contribution of Western and Chinese ancestry to Beijing Black pigs. These findings enable us to propose a reliable and sustainable strategy for the conservation and improvement of Beijing Black pigs.

## Data Availability

The datasets presented in this study can be found in online repositories. The names of the repository/repositories and accession number(s) can be found in the article/Supplementary Material.
