# Supplementary material for: Population Genetic Structure and Selection Signature Analysis of Beijing Black Pig
Source: Front Genet. 2022 Mar 24;13:860669. doi: 10.3389/fgene.2022.860669 (PMC8987279; doi:10.3389/fgene.2022.860669)
Supplement: Supplementary file 1 [file DataSheet1.docx]

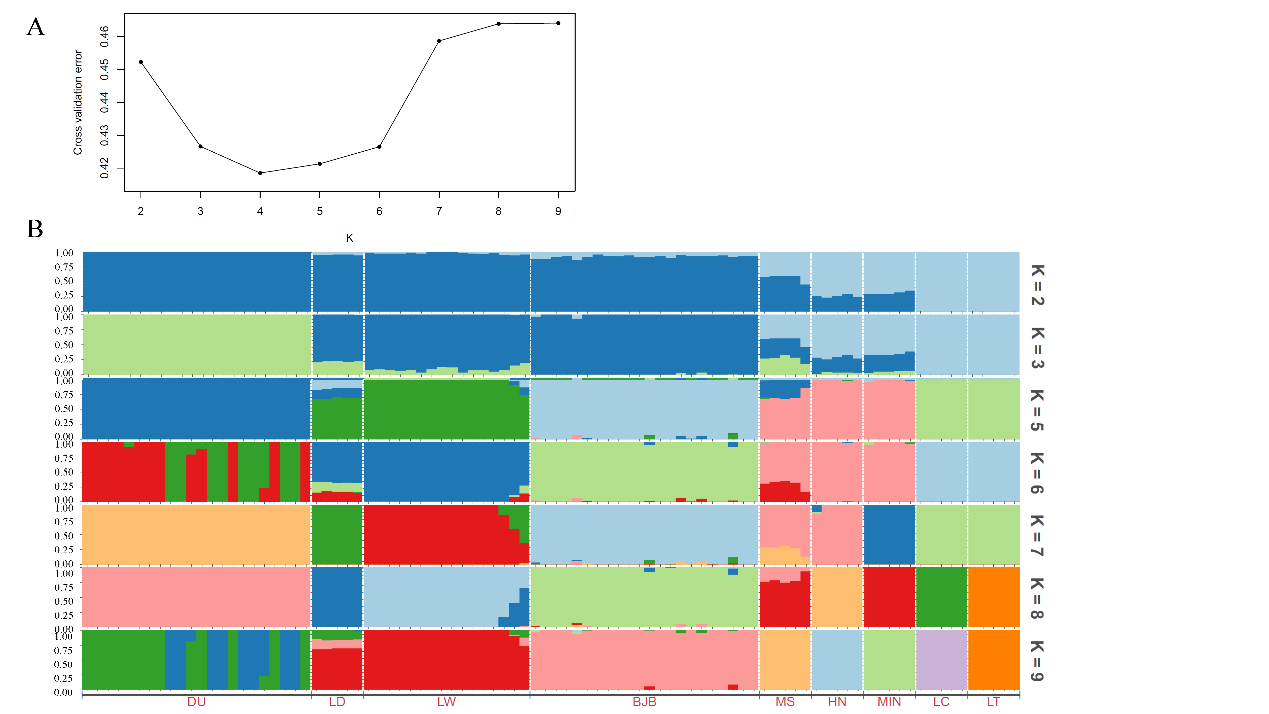


**Figure S1 |** ADMIXTURE analysis was performed to estimate the optimum number of clusters (k) in the data set. **(A)** Cross validation errors for diverse k values. As shown, k = 4 minimizes the cross-validation error. **(B)** Ancestry of each sample using k = 2-3, 5-9 clusters.


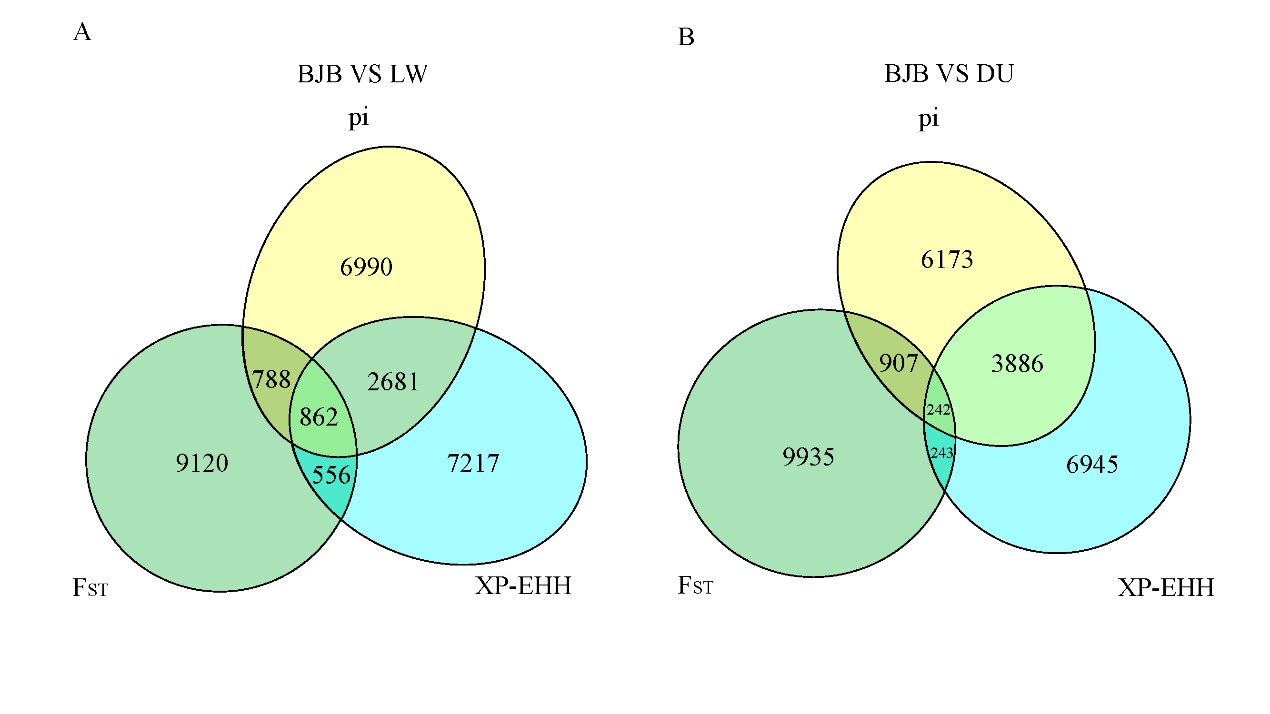


**Figure S2 | (A)** Venn diagram shows the overlap in the number of candidate regions detected by three methods between Beijing black (BJB) and Large white (LW) pigs. **(B)** Venn diagram shows the overlap in the number of candidate regions detected by three methods between Beijing black and Duroc (DU) pigs.
